# Heparan Sulfate Proteoglycans Biosynthesis and Post Synthesis Mechanisms Combine Few Enzymes and Few Core Proteins to Generate Extensive Structural and Functional Diversity

**DOI:** 10.3390/molecules25184215

**Published:** 2020-09-14

**Authors:** Thibault Annaval, Rebekka Wild, Yoann Crétinon, Rabia Sadir, Romain R. Vivès, Hugues Lortat-Jacob

**Affiliations:** Institut de Biologie Structurale, UMR 5075, University Grenoble Alpes, CNRS, CEA, 38000 Grenoble, France; Thibault.Annaval@ibs.fr (T.A.); Rebekka.Wild@ibs.fr (R.W.); Yoann.Cretinon@ibs.fr (Y.C.); Rabia.Sadir@ibs.fr (R.S.)

**Keywords:** proteoglycan, glycosaminoglycan, heparan sulfate, glycosylation, biosynthesis, biosynthesis and post synthetic enzymes

## Abstract

Glycosylation is a common and widespread post-translational modification that affects a large majority of proteins. Of these, a small minority, about 20, are specifically modified by the addition of heparan sulfate, a linear polysaccharide from the glycosaminoglycan family. The resulting molecules, heparan sulfate proteoglycans, nevertheless play a fundamental role in most biological functions by interacting with a myriad of proteins. This large functional repertoire stems from the ubiquitous presence of these molecules within the tissue and a tremendous structural variety of the heparan sulfate chains, generated through both biosynthesis and post synthesis mechanisms. The present review focusses on how proteoglycans are “gagosylated” and acquire structural complexity through the concerted action of Golgi-localized biosynthesis enzymes and extracellular modifying enzymes. It examines, in particular, the possibility that these enzymes form complexes of different modes of organization, leading to the synthesis of various oligosaccharide sequences.

## 1. Introduction

Protein glycosylation, which takes place in thousands of proteins, is considered as one of the major post-translational modifications in proteins. Modifications with N- or O-glycans has important effects on protein folding, conformation, distribution and stability and serves a large number of purposes including protein-ligand binding, protein folding and stability, cell-cell communication and signaling or pathogen recognition and infection [1]. Amongst these, a very small set of proteins, known as proteoglycans (PG), represent a specific and important class of glycoproteins [2]. In addition to being glycoproteins, as defined above, they are covalently linked to polysaccharides of the glycosaminoglycan (GAG) family, which includes heparin (Hep), heparan sulfate (HS), dermatan sulfate (DS), keratan sulfate (KS) or chondroitin sulfate (CS). The number of proteoglycans is fairly limited: one for HepPGs, approximately 20 for HS- and CS-PGs and 8 for KSPGs. However, tremendous diversity characterizes this family of glycoproteins, due to the variable number and the combination of GAG chains found in each PG and, most importantly, the structural diversity of the GAG chains themselves (see below). Another remarkable feature of PGs is their presence at the cell surface, within the pericellular environment and in the extracellular matrix [2]. These very different locations, combined with their structural diversity, enable them to participate in a large variety of cellular processes. Importantly, whereas PG’s core protein directs PG localization, their GAG chains appear to be responsible for most of their biological functions. The present review will focus on heparan sulfate proteoglycans (HSPGs), whose GAG chains are the most complex and information-rich polysaccharide.

**Figure 1:**
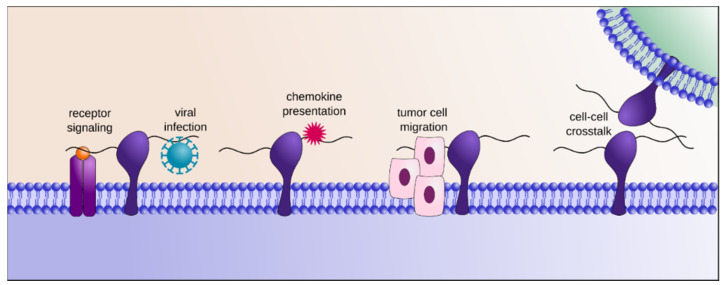
Heparan sulfate proteoglycans (HSPGs) contribute to the formation of the glycocalyx at the cell surface and are ubiquitously present in the extracellular matrix, where they interact with a myriad of different HS binding proteins. In doing so, they integrate the flow of information that circulates in-between cells and upstream signaling that is mediated by the cytokines/growth factors-receptor interaction. They are also further involved in chemokines/morphogens gradient stabilization and presentation, cell migration and adhesion or microbial adsorption, thereby regulating many fundamental biological processes.

HS exerts its biological functions by interacting with a vast array of protein ligands, such as growth factors, cytokines, chemokines, morphogens, receptors, adhesion molecules, viral envelopes etc. (Figure 1), thereby controlling their transport, local concentration, clearance, stability and modulating their conformation and bioactivity [3]. For example, HS provides templates to assemble active supramolecular complexes such as growth factor/growth factor receptor thus facilitating signal transduction [4,5], protects cytokines against proteolysis [6], mediates the formation of chemokine gradients along which cells can migrate directionally [7], induces protein conformational changes [8], provides cell surface docking sites for pathogens [9] etc. As such, it interferes with and controls most biological processes, including cell proliferation, migration and development, inflammation and immune response, lipid metabolism, angiogenesis, matrix assembly, tissue repair or host-pathogen interaction [10].

**Figure 2:**
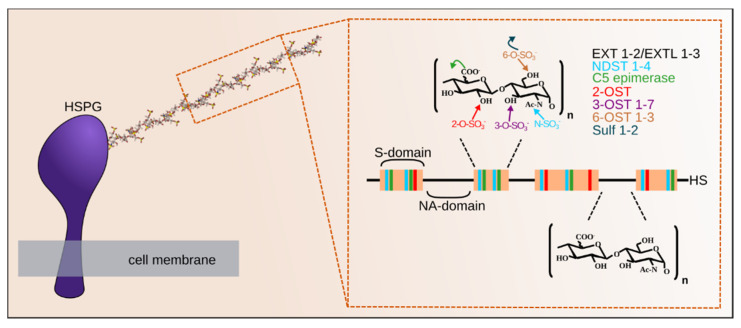
Heparan sulfate (HS) is a linear polysaccharide in which a glucuronic acid- *N-*acetyl glucosamine (GlcA-GlcNAc) repeat is polymerized by the exostosins (EXTs) enzymes and modified in restricted domains (S-domain, in red) through the action of the *N-*deacetylase/*N-*sulfotransferase enzymes (NDSTs), C5 epimerase, 2-, 3- and 6-*O-*sulfotransferases (OSTs). These enzymes, localized within the Golgi apparatus, generate up to (the theoretical number of) 48 different disaccharides (23 of which have been identified yet), the combination of which gives rise to hyper variable structures spaced apart by more regular *N-*acetylated (NA)-domains (in black). Bound to a core protein to form HSPG, the proteoglycan is exported to the cell surface or the extracellular matrix, where Sulfs can further modify the polysaccharide. Both the sulfated (S)- and NA- domain organization of the HS chains and the extensive variability within the S domains provide the polysaccharide with distinct binding sites for its various protein ligands.

Consistently with its large interactome [11] and extensive functional repertoire, HS is characterized by a unique level of structural complexity, which is imprinted onto the polysaccharide backbone during biosynthesis by Golgi-localized enzymes, and through post synthetic extracellular processes. HS consists of a repeating disaccharide unit, comprising of a *N-*acetyl glucosamine (GlcNAc) and a glucuronic acid (GlcA), initially polymerized by the Exostosin 1 and 2 (EXT1 and EXT2) enzymes [12], with the participation of EXT-like proteins (EXTL). During biosynthesis, the polymer that typically comprises 50 to 400 monosaccharide units, is variously modified by interdependent enzymatic reactions that do not occur uniformly along the chain (Figure 2). The GlcNAc residues can be first *N-*deacetylated, followed or not by re-*N-*sulfation, catalyzed by the *N-*deacetylase/*N-*sulfotransferase enzymes (NDSTs), giving rise to unsubstituted glucosamine (GlcN) or *N*-sulfated glucosamine (GlcNS). Remarkably, this occurs in contiguous stretches of usually 3–6 disaccharides (known as S-domains). Within these domains, the GlcA can be converted into iduronic acid (IdoA) by a C5-epimerase and the chain can be further modified by 2-, 6- or 3-*O-*sulfotransferases (OSTs) catalyzing *O-*sulfations at some of the C6 and C3 positions of the GlcN and of the C2 positions of the IdoA (and more rarely GlcA) residues [13,14,15]. Once exported to its final extracellular position, HS can be further modified by the sulf enzyme, catalyzing specific GlcN 6-*O-*desulfation [16].

A wealth of studies reported that HS structural diversity is tightly controlled and dynamically regulated at the cell type and tissue level, but also during development and in pathological conditions [17,18,19,20,21,22], whereas HS from different sources shows extensive structural variability, analysis of HS from various mammalian organs has revealed organ-specific differences that appeared reproducible within a given species. Similarly, immunohistochemical analyses using antibodies selectively recognizing distinct HS epitopes revealed different patterns of individual HS motifs within tissues. These observations reflect differences in HS domain composition, sequence and/or general organization of the chain and support the view that, during biosynthesis, oligosaccharide motifs characterized by specific sulfation and epimerization patterns can be assembled to display the necessary information for protein ligand recognition. However, unlike any other biopolymers (DNA, RNA, polypeptides), HS biosynthesis is a non-template driven process and it is built in the absence of genetically encoded information. Therefore, how the fine structure of HS is determined remains poorly understood.

After briefly describing HSPGs, this manuscript will review the current knowledge on the biosynthesis and post synthesis machineries that shape the HS structure, focusing on the concept that the enzymes involved in these processes interact with each other, and cross control their biological activities, hence the final structure of their product.

## 2. Heparan Sulfate Proteoglycans

### 2.1. Pericellular and Extracellular Matrix HSPGs

PGs are one of the main constituents of extracellular matrices (ECM). Matrix PGs generally correspond to large modular proteins, carrying numerous chains of GAGs. Most ECM PGs are of the chondroitin/dermatan sulfate proteoglycan (CS/DSPG) type, but four major HSPGs have been identified:

-Perlecan. Perlecan is expressed in the basement membrane of epithelial and endothelial cells, as well as in cartilage [23]. Its 470 kDa protein core includes five modules, domain I carrying three GAG chains (generally HS). Perlecan binds, via its protein modules or its GAG chains, to various ECM proteins and cell adhesion molecules, such as integrins, fibroblast growth factors (FGFs), laminin-1, fibronectin, collagen IV, etc. Through these interactions, perlecan plays a major role in the basement membrane architecture and integrity, as well as in physiological and pathological processes, such as atherosclerosis, angiogenesis and cancer [2,24]. In this context, it is worth noting that cleavage by proteolysis of the C-terminal region of perlecan leads to the release of endorepellin, a peptide with potent anti-angiogenic properties [25]. Perlecan-deficient mice show severe, often lethal chondrodysplasia [26]. In humans, mutations in the HSPG2 gene encoding perlecan lead to various genetic disorders. Mutations that completely abrogate perlecan expression or secretion into the ECM cause severe neonatal lethal Silverman–Handmaker type of dyssegmental dysplasia [27]. In contrast, mutations affecting perlecan domain V or reducing perlecan level of expression have been associated with mild Schwartz–Jampel syndrome, a pathology characterized by myopathy, chondrodysplasia and abnormal neuromuscular junctions due to an absence of acetylcholinesterase activity [28].

-Agrin. Agrin is a 225 kDa modular HSPG that is widely expressed in basement membrane. Agrin carries at least three HS chains located in the *N-*terminal region of the protein, which interacts with a variety of ligands, including FGFs, α-dystroglycan, laminins, neural cell adhesion molecule (*N-*CAM) and β1-integrins [29,30]. Alternative splicing of the agrin gene leads to the synthesis of two isoforms with very distinct biological properties: a type II transmembrane protein (TM-agrin) and a secreted extracellular form [31]. Agrin plays an important role in neuromuscular junction post-synaptic membranes, where it induces the clustering of acetylcholine receptors (AChRs) [32]. Similarly, agrin has been proposed to take part in the regulation of the so-called “immunological synapse”, by promoting membrane lipid microdomains reorganization and T-cell receptor clustering [33], and to the “viral synapse” that allows transcytosis of HIV across the epithelial cell monolayer [34].

-Collagen XVIII. Collagen XVIII is a homotrimer organized in a triple helix composed of 10 interspersed collagen-like domains. It harbors three HS chains, as well as endostatin modules located within a C-terminal non-collagenous region. Collagen XVIII is ubiquitously expressed and is particularly found in the basement membranes of vascular and epithelial tissues. Functionally, collagen XVIII (and its endostatin domains) is involved in the control of angiogenesis and wound healing [35]. It exhibits anti-atherosclerosis properties [36], and mediates leukocyte influx in renal ischemia/reperfusion [37]. Finally, it has been associated with vascular amyloid depositions during Alzheimer’s disease [38]. In humans, mutations in the gene encoding collagen XVIII have been associated with Knobloch syndrome, characterized by severe ocular defects including acute myopia and retinal degeneration [39], as well as hypertriglyceridemia [40].

-Testican/SPOCK. This family comprises three members originally termed testicans (1–3) and recently renamed SPOCK (SPARC/Osteonectin CWCV and Kazal-like domain) based on amino acid sequence homology. SPOCKs are modular HSPGs, with a C-terminal domain five comprising of two HS attachment sites [2]. Testicans were originally isolated from seminal fluid, but they are actually expressed almost exclusively in the central nervous system (CNS). SPOCK-1 is present in the post-synaptic area of the hippocampus [41], SPOCK-2 is found in various neuron-rich regions in the cerebellum and brain, as well as in lung and testis [42] and SPOCK-3 is ubiquitously expressed within the CNS [43]. SPOCKs are primarily involved in the control of neuronal development. In line with this, a mutation of the gene encoding SPOCK-1 has been associated with CNS developmental delay, resulting in microcephaly associated with mental retardation [44]. Likewise, SPOCK-3 (knock out) KO mice showed defects in CNS development and abnormal behavior [45].

### 2.2. Cell-Surface HSPGs

The majority of PGs found at the cell surface are HSPGs. Two main families of cell-surface HSPGs are the syndecans and glypicans, which can be distinguished by the nature of their protein core and their mode of anchoring to the membrane.

-Syndecans. Syndecans are a family of four distinct proteins, termed syndecan-1–4 according to their order of discovery. They are type I transmembrane proteins, consisting of a short intracellular C-terminal sequence, a transmembrane region and an extracellular domain. The extracellular domain is the most variable region of the protein, with only 10–20% sequence similarity between syndecans [46]. However, some sequence features remain highly conserved, such as a cluster of basic amino acids close to the transmembrane domain, the recognition motifs of proteases responsible for syndecan ectodomain shedding and the GAG attachment chains. Remarkably, the nature, number and position of GAG chains on syndecans is unvarying across species, thus suggesting the essential role of the ectodomain in the presentation and distribution of GAG chains in a well-defined spatial arrangement. Syndecans-1 and -3 display GAG attachment sites in two distinct regions located at both ends of their ectodomain, and are hybrid proteoglycans carrying both HS and CS/DS chains [47]. For the former, CS/DS chains are mostly found in the membrane proximal attachment sites. In contrast, syndecans-2 and -4 only feature the *N-*terminal end GAG attachment sites and are exclusive HSPGs.

Contrary to the ectodomain, syndecan transmembrane and cytoplasmic regions are very homologous (60–70% sequence identity).

The transmembrane domain mostly consists of hydrophobic amino acids and exhibits a GXXXG motif (where G is glycine and X is an undefined amino acid) involved in the homo or heterodimerization of syndecans, and in interactions with membrane receptors [48]. The cytoplasmic domain is characterized by the presence of two highly conserved regions, domains C1 (near the transmembrane domain) and C2 (near the C-terminal end), separated by a variable V region, exhibiting poor homology between syndecans, but high interspecies conservation. The V region may be important for specific functions of syndecans. For instance, protein kinase C (PKC) and phosphatidyl inositol di-phosphate (PIP2) interact with the V region of syndecan-4 during the formation of focal adhesion complexes [49,50]. In contrast, C1 and C2 domains may be involved in the biological functions shared by all syndecans. As such, domain C1 interacts with proteins of the cortactin/Src signalling pathway [51], and domain C2 features a EFYA tetrapeptide binding motif for PDZ domain-containing proteins, such as syntenin [52] and CASK/Lin-2 [53]. Although syndecans are present at the surface of most mammalian cells, their expression is tissue and developmental stage specific [54,55]. Syndecan-1 is mainly found in epithelial cells and circulating malignant cells, while syndecan-2 is present in endothelial cells, fibroblasts, and is particularly abundant in the liver [46,56,57]. Syndecan-3 is only found in neuronal tissues [54,58,59], whereas syndecan-4 is the most broadly expressed [54,60].

-Glypicans. Originally identified in human pulmonary fibroblasts [61], glypicans are found in a wide range of cells and tissues. Glypicans are highly conserved proteins (more than 90% homology between species), characterized by a cysteine-rich 63kDa extracellular protein core linked to the plasma membrane via a glycosyl-phosphatidyl inositol (GPI) anchor. The glypican family comprises six members: glypican-1 [61], glypican-2 or cerebroglycan that is mostly expressed during the development of the nervous system [62], glypican-3 or OCI-5 [63], glypican-4 or K-glypican [64], glypican-5 [65] and glypican-6 [66,67]. Glypicans are strict HSPGs, with the exception of glypican-5, which also carries CS/DS chains [68,69]. Although these proteins lack transmembrane and cytoplasmic domains, glypicans have been shown to be involved in the cellular response of many growth factors and morphogens [70,71]. Some biological properties of glypicans could be explained by their GPI-like membrane anchoring, which allows their localization (and that of their ligands) in raft-specialized membrane micro-domains [72,73,74]. Additionally, glypicans can be released from the cell surface through an original shedding mechanism involving the cleavage of the GPI anchor by an extracellular lipase [75,76]. Expression of glycpicans is spatially and temporally regulated [77], and changes in glypican expression have been observed in different cancers [78,79,80]. Finally, a mutation in the glypican-3 gene has been associated with Simpson–Golabi–Behmel syndrome [81,82,83].

-Other cell-surface HSPGs. Apart from the syndecan and glypican families, other cell-surface proteins have been shown to bear HS chains. This is the case for the type III TGFβ receptor, also called betaglycan [84,85], which can bind simultaneously to TGFβ via its protein core and to FGF-2 via its HS chains [86]. Similar to syndecans, the betaglycan cytoplasmic region features a PDZ domain binding motif [84], and its extracellular region can be released upon shedding [87]. Betaglycan has been associated with epithelial–mesenchymal transformation during embryogenesis, as well as tumor progression [88,89].

HS chains have also been found on the CD44E isoform (or epican), resulting from alternative splicing of the CD44 gene [90]. CD44E is expressed in monocytes, where it binds a number of ligands, including FGF-2, VEGF and HB-EGF, but not chemokines, such as MCP-1 or IL-8 [91]. In contrast, CD44E in endothelial cells has been associated with the binding and promotion of pro-inflammatory chemokine MIP-1β [92]. CD44E is associated with inflammation, wound-healing and tumor metastasis migration [93,94,95].

Finally, part-time HSPGs include neuropilins [96], as well as a particular isoform of the FGF receptor, FGFR2-IIIb [97,98].

### 2.3. A Unique Intracellular PG: Serglycin

-Serglycin. Serglycin was the first PG to be cloned and remains so far the only known intracellular PG [99]. Serglycin is so called, as its 17 kDa protein core features multiple serine-glycine repeats [100]. Originally discovered in the secretion vesicles of hematopoietic cells, serglycin has since been identified in fibroblasts, smooth muscle cells, chondrocytes, endothelial cells and embryonic stem cells [101,102,103]. A particular feature of serglycin is that the nature of its associated GAG chains varies amongst cells. In circulating cells, such as lymphocytes, platelets, and monocytes, serglycin is associated with low-sulfated chondroitin 4-sulfate (CS-A) chains. In contrast, serglycin from hematopoietic cells, including mucosal and bone marrow mast cells and activated monocytes and macrophages, exhibits highly sulfated CS-E or DS chains. Finally, serglycin produced by connective tissue mast cells harbors heparin chains and may thus be classified as an HSPG [100,103].

In connective tissue mast cells, heparin-bearing serglycin plays a critical role in the storage and packaging of secretory granule components to be released upon inflammation, including proteases, histamine or serotonin [103,104]. In agreement with this, serglycin KO mice displayed secretory granule storage defects [105,106], a phenotype shared with cells deficient in heparin-synthetizing NDST2 enzyme [107]. Serglycine is also found in secretory granules from other inflammatory cells and in platelets, where it is involved in the storage of growth factors, cytokines, chemokines and granzyme B [103,108,109,110]. Secretion of these molecules in complex with serglycin further promotes their activity, as serglycin may protect them against proteolysis and facilitate their presentation to target cells. As such, serglycin has been associated with inflammatory diseases and platelet disorders [111]. Finally, serglycin is also found in a number of cancer cells [111], including breast cancer cells, for which its expression has been correlated with aggressive malignant phenotype [112].

## 3. The Gagosylation Process Initiation and the Golgi Apparatus

Gagosylation is initiated onto a PG core-protein in the Golgi apparatus or at the endoplasmic reticulum (ER)-Golgi interface. The Golgi structure and organization are relatively well defined. This organelle has a unique morphology and is constituted of cisternae, vesicles and tubular structures. The ultrastructural images of the Golgi were first obtained by electron microscopy (EM) approaches [113]. Further development in EM techniques including electron-tomography-enabled 3D imaging of the Golgi apparatus [114]. The cisternae (four to eight), consisting of a pile of flattened, stapled pouches, form the Golgi stack. This structure, referred to as the compact zone of the Golgi, can be divided in three separate compartments: the cis-, medial-, and trans- cisternae [115]. The cis- and trans-Golgi networks (CGN and TGN) are not comprised in the Golgi stack. The CGN, also named ER-Golgi intermediate compartment (ERGIC), is a complex membrane system between the ER and the Golgi, receiving cargo from the ER. The TGN is facing the plasma membrane and is the main site for the sorting of molecules for post-Golgi export [116]. The Golgi elements are interconnected by a tubular network and surrounded by numerous vesicles. There are two types of vesicle coat protein, COPI and COPII, with different functions. COPI vesicles are involved in the retrograde transport, (from cis-Golgi and ERGIC compartment to the ER) and between Golgi cisternae [117]. COPII mediates traffic from the Golgi to the ER (anterograde transport) [118,119].

As mentioned above, HS biosynthesis enzymes are primarily localized within the Golgi apparatus (Table 1). Most of them belong to the type II transmembrane proteins. They feature a N-terminal peptide that serves as a membrane signal anchor sequence and protrudes in the cytoplasm, a single transmembrane domain and a larger C-terminal domain, present within the lumen of the Golgi compartment and exhibit the catalytic activity. GAG biosynthesis thus takes place in the lumen of the Golgi apparatus and starts with the synthesis of a tetrasaccharide primer linked to the serine residue of the PG core-protein. There is no formal consensus sequence that defines gagosylation sites, except that a glycine amino acid occurs immediately on the carboxy-terminal side of the modified serine and two acidic residues are present in close proximity. This linkage tetrasaccharide is synthesized by the sequential stepwise addition of a xylose (Xyl), two galactoses (Gal) and a glucuronic acid (GlcA) to the target serine residue, carried out by the action of two *O*-xylosyltransferases (XylT-1 or XylT-2), two galactosyltransferases: β1,4-galactosyltransferase 7 (GalT-1) and β1,3-galactosyltransferase 6 (GalT-2) and a β1,3 glucuronosyltransferase I (GlcAT-1) [120,121,122]. Several studies, using confocal microscopy and specific Golgi markers, suggest that the enzymes involved in the formation of the linkage region, XylT-1, XylT-2, GalTI and GlcATI, localized independently in the cis medial-Golgi element [123,124].

Following the assembly of the tetrasaccharide linker, the transfer of either a αGlcNAc or a βGalNAc on the GlcA terminal residue initiates the further polymerization of HS or CS chains, respectively [130,131]. Structural analysis of the tetrasaccharide linker has revealed that the Xyl residue can be phosphorylated at C2 position, whereas the two Gal residues can be sulfated at C4 or C6 position. Xylose C2 phosphorylation, which has been observed in both HS and CS, seems to be a transient process whereby FAM20B, the kinase involved in xylose phosphorylation, enhances the formation of the tetrasaccharide linker [132,133], while a 2-phosphoxylose-phosphatase efficiently removes the phospho group, just after the tetrasaccharide linker synthesis and just before polymerization of the GAG chains starts. Indeed, at least in vitro GAG polymerization does not occur when the Xyl residue of the tetrasaccharide linkage region is phosphorylated. The EXT-like protein EXTL2 can add a GlcNAc residue to the phosphorylated tetrasaccharide linker; however, this cannot be further elongated and therefore GAG chain elongation is terminated [134]. Interestingly however, the 2-phosphoxylose-phosphatase activity is enhanced by GlcAT-I [135], the enzyme adding the fourth linker sugar, with which the phosphatase forms a hetero-oligomeric complex [136]. Temporary modifications of the tetrasaccharide-linker thus control GAG chain elongation and formation in a complex fashion. Sulfation of the linker region has never been observed for HS and heparin chains, and is limited to the CS/DS pathway, for which it stimulates biosynthesis [137].

## 4. Heparan Sulfate Elongation

The polysaccharide chain elongation is carried out by the glycosyltransferases EXT1 and EXT2, which catalyze the alternating addition of a glucuronic acid (GlcA) and GlcNAc residue [125,138]. EXT1 and EXT2 were both shown to exhibit GlcA and GlcNAc transferase activities in vitro [126,139]. It was further shown that EXT2 has only a low glycosyltransferase activity itself, but it can increase the activity of EXT1 in vitro [140]. In vivo, the two proteins seem to have different functions and cannot substitute for each other [140,141]. Interestingly, EXT1 and EXT2 physically interact in co-immunoprecipitation assays and form a complex in the ER before trafficking to the cis-Golgi compartment where they are functionally active [140,142]. The dependence of the dynamic trafficking, localization and interaction of these enzymes on HS biosynthesis has also been demonstrated in drosophila, where retini, the insect homolog of Golgi phosphorylated protein (GOLPH)3, was shown to interact with the EXT, by co-immunoprecipitation. Overexpression or knock-out of GOLPH3 led to the re-localization of the EXT to the cis- or trans-Golgi network, respectively, and resulted in impaired HSPG formation [143]. Interaction between EXT1 and EXT2 was further demonstrated in vitro using size exclusion chromatography [139]. However, the molecular basis of complex formation and the catalyzed chain elongation reaction remains to be elucidated.

Chain elongation is a central step in HS biosynthesis and EXT1 and EXT2 activity is regulated through several mechanisms, including changes in gene expression levels, phosphorylation or sulfation of the tetrasaccharide linker region, protein–protein interaction with other HS biosynthesis enzymes and, as mentioned above, localization of the enzymes to different Golgi compartments.

For example, gene expression of EXT1 was found to be abrogated in human cancer cells and epigenetic loss of EXT1 in leukemia and non-melanoma skin cancer was linked to CpG island hypermethylation, resulting in loss of HS [144]. In addition, EXT1 can be regulated by microRNAs. High miR-655 expression levels, as found in acute lymphoblastic leukemia, were correlated to low EXT1 expression and thus reduced apoptosis and increased cell growth [145]. Whether cells modulate EXT1 and EXT2 gene expression levels under physiological conditions to generate specific HS structures has not been investigated yet.

## 5. Heparan Sulfate Maturation

### 5.1. GlcNAc De-N-Acetylation/Re-N-Sulfation

The nascent polymer is next modified by a bifunctional enzyme, the *N-*deacetylase-*N-*sulfotransferase (NDST) that exists under four isoforms. The NDSTs catalyze the de-*N-*acetylation and re-*N-*sulfation of the GlcNAc units of the HS chain. Deacetylation of GlcNAc, at position two, leads to the formation of GlcN, whose free amine is usually re-sulfated into GlcNS using 3′-phosphoadenosine-5′-phosphosulfate (PAPS) as sulfate donor [146]. This reaction is the starting point for maturation and the presence of GlcNS is essential for the following modifications [147]. However, this conversion is not exhaustive along the polymer and leads to the formation of *N-*sulfated domains (referred as S-domains) inserted within the *N-*acetylated regions (NA-domains) [148,149]. Modification appeared to occur in a processive manner [146], from the non-reducing end to the reducing end [150], but how the starting point and the extent of the modification are determined remain poorly known.

Four isoforms of NDST have been identified [151,152,153,154,155]. Knockout of these genes in mice leads to different phenotypes [107,156,157,158,159,160]. NDST1 knockout leads to neonatal death caused by immaturity of type II pneumocytes, and HS analysis shows a drastic reduction in GlcNS content [156,157]. Contrary to NDST1, the inactivation of NDST2 is not lethal but leads to a reduction of mast cells in the connective tissue and to a reduced HS chain length [107,158]. Finally, knockouts of NDST3 and NDST4 do not cause major phenotype changes. Mice are viable and fertile and only the lack of NDST3 induces moderate undersulfation of HS [159,160]. These observations suggest that the four isoforms act at different times of the development and exhibit different catalytic activities. Expression analysis of the NDSTs, as measured by mRNA quantification, shows that NDST1 and NDST2 are broadly expressed in a wide range of tissues in contrast to NDST3 and NDST4, which are only expressed in the brain and during embryonic development [155,161].

Multiple efforts have been made to study both the deacetylase and sulfotransferase enzymatic activities of each isoform. Each NDST exhibits the two activities with the exception of NDST4, which lacks the *N-*deacetylase’s one [155,162,163,164]. For NDST one and two, the *N-*deacetylase reaction appears to be the limiting step for the whole NDST activity [155]. In contrast, NDST3 features a poor *N-*sulfotransferase activity [154], and can thus give rise to unsubstituted GlcN residue in HS, whose biological importance has been highlighted in L-selectin- or cyclophilin B dependent integrin- mediated cell adhesion, recognition [165,166] or heparanase inhibition [167]. Differences in domain activities, as well as potential unknown factors, lead to variability in S-domain length, which can be exacerbated by the predominance of an isoform within a tissue [107]. PAPS concentration was also mentioned as a promotor of NDST activity and of S-domain length [146]. Finally, *N-*deacetylase activity is increased in the presence of its *N-*sulfotransferase partner on the same polypeptidic chain and the use of NDST variants exhibiting *N-*deacetylase and *N-*sulfotransferase activities separately altered HS sulfation pattern [163]. This information highlights the cooperation of the two domains during the enzyme reaction, possibly helped by physical contact. No structure of NDST has been determined yet. Only the structure of the *N-*sulfotransferase domain from NDST1 has been solved by X-ray crystallography [168], therefore the mechanism by which the two domains cooperate, is not understood at the molecular level.

Interestingly, it has been also reported that over-expression of EXT2 in HEK293 cells increased both NDST expression and activity in cells, while over-expression of EXT1 has the opposite effect. Such observations have been tentatively explained by hypothesizing that NDST1 could depend on EXT2 for its transport to the proper Golgi compartment and EXT1, by binding to EXT2, would out compete NDST1 from the complex. Supporting this view, co-immunoprecipitation experiments demonstrated a physical interaction of EXT2 and NDST1 [169].

### 5.2. GlcA C5 Epimerization

Epimerization of GlcA into IdoA is carried out by a single isoform C5-epimerase [170]. This enzyme catalyzes the reversible abstraction and re-addition of the C5 proton of GlcA immediately adjacent to a GlcNS [171], indicating that this step occurs after the NDST-mediated process [172,173]. The crystal structure of the free human C5-epi was recently reported as well as that of an inactive mutant bound to a (GlcA-GlcNS)n substrate or a (IdoA-GlcNS)n product. It revealed the presence of an extensive network of specific interactions that explained in particular the absolute requirement of *N-*sulfate groups vicinal to the epimerization site for substrate binding [174]. The structures show that the GlcA/IdoA rings into the active site cleft are highly constrained, at the epimerization site, into almost identical boat conformations, giving a mechanistic rationale for the reversible nature of the reaction. The structure of the zebrafish C5-epimerase has also been solved in complex with heparin [175], a highly sulfated form of HS, in which most IdoA are 2-*O* sulfated. In this structure, the IdoA2S shows a large positional difference, compared to its non-sulfated counterpart of the authentic substrate, and is shifted seven Å away from the epimerization site, explaining that the 2-*O* sulfate group blocks the back epimerization of the residue [176]. Interestingly, several studies indicated that 2-OST and C5-epi co-localize in the Golgi apparatus [123] and physically interact [177], thus suggesting that these enzymes form a complex allowing efficient coupling of 2-*O*-sulfation with C5 epimerization, and thereby generating extended sequences of contiguous GlcNS-IdoA2S. IdoA residues (and IdoA2S, see below), due to their conformational flexibility, contribute significantly to protein recognition [178]. Lack of the C5-epimerase gene in mice is lethal, inducing kidney, lung and skeletal defects [179]. These phenotypes are presumably the consequence of the loss of flexibility of HS in the absence of IdoA unit [180] and reduced protein binding capacity [181], as well as a reduced level of HS 2-*O*-sulfation [182], due to GlcA being a poorer substrate of 2-OST than IdoA.

### 5.3. GlcA/IdoA and GlcN O-Sulfation

HS then undergoes a series of *O*-sulfation catalyzed by 2-OST, 6-OST and 3-OST, which also use PAPS as a sulfate donor.

2-OST exists as a single isoform and catalyzes the transfer of a sulfo group onto the 2-OH position of uronic acids. Additionally, 2-OST preferentially targets IdoA, but also GlcA, giving rise to IdoA2S or GlcA2S. Structural studies showed that the IdoA residue within the enzyme’s active site adopts a ^4^C_1_ conformation, rather than the more common ^2^S_0_ or ^1^C_4_ conformations for this residue. Interestingly, such a conformation is the only one observed for GlcA, suggesting that the active site of 2-OST can accommodate a single conformation to catalyze *O*-sulfation of either IdoA or GlcA. However, 2-OST, has a five-fold higher affinity for IdoA than for GlcA [183]. Consequently, IdoA2S is a common motif found in HS, in particular within the S-domains, and is frequently involved in protein binding. It is thus key to the polymer activity. Its critical function has been highlighted in several studies, as well as by the large developmental defects and premature death observed in 2-OST knockout mice [179,184].

The physiological importance of GlcA2S is not fully understood, but it has been detected in relatively high quantities within certain tissues or cells, such as in the human cerebral cortex or in hepatocytes [183,185,186]. As mentioned above, purified recombinant soluble 2-OST interacts with C5-epi [177], which may provide a mechanistic explanation of the higher proportion of IdoA2S versus GlcA2S.

A substrate specificity analysis showed that the 2-OST acceptor residues must be flanked by two GlcNS, consistently with the observation that *N-*sulfo groups mediated many contacts with several amino acids of the enzyme [187]. Oligosaccharides containing a 6-*O*-sulfate group also bound strongly to the 2-OST active site, but the 6-*O*-sulfate moiety appeared to occupy the PAPS binding site and thus inhibited the 2-*O* sulfation reaction [188]. Altogether, these data suggest 2-*O*-sulfation occurs prior to 6-*O*-sulfation to generate IdoA2S-GlcNS6S sequences and that the 2-OST exhibits multiple structural features to recognize its substrate sulfation pattern.

6-OST, which exists under three isoforms, transfers a sulfate group to the six position of either GlcNAc or GlcNS, giving rise to GlcNAc6S or GlcNS6S, respectively. Although studies of 6-OSTs did not reveal major differences in isoform substrate specificities, 6-OST1 was found to preferentially target IdoA-GlcNS sequences, generating 6-*O-*sulfated domains with low 2-*O-*sulfate content, 6-OST2 was more active towards 2-*O-*sulfate containing motifs and 6-OST3 displayed an intermediate substrate specificity [189,190]. The absence of strict substrate specificity for 6-OSTs could possibly explain the compensation of sulfate loss observed upon deletion of the 2-OST gene, through an increase in the overall six sulfation content of the polysaccharide [184]. Nevertheless, 6-OST isoforms can be distinguished by specific spatio-temporal distribution in tissues and during development [191,192,193]. Such differential expression may thus provide HS sequences with different binding motifs and contribute to the specific activities of the polysaccharide. Similarly to 2-OST, 6-OST1 was shown to interact with C5-epi (in zebrafish), although to a lesser extent, but the functional consequences of this interaction have not been analyzed yet [175].

Finally, HS chains can be further modified by 3-OSTs, which add a sulfo group to the three position of GlcN units [194]. With seven isoforms, 3-OSTs represent the largest family among HS sulfotransferases. Surprisingly, the occurrence of 3-*O-*sulfation is poorly known and is believed to be low: around 1% in endothelial cells [195] and 5 to 10% in Reichert’s basement membrane or follicular fluid [196,197]. The 3-OST family has been divided in two subgroups, based on structural homology, whose activities are involved in the generation of binding sites for antithrombin and for the glycoprotein gD of type I herpes simplex virus, respectively, and are often referred as “AT-type” and as “gD-type” [198,199]. The former comprise 3-OST-1 and 5, and the latter 3-OST-2, 3a, 3b, 4 and 6. Additionally, 3-OST1 preferentially targets GlcN units with a non-reducing end linked to a GlcA devoid of 2-*O-*sulfate. In contrast, gD-type 3-OSTs preferentially target GlcN units associated with a 2-sulfated IdoA. Finally, 3-OST5 catalyzes the sulfation of GlcN irrespective of 2-O sulfation. Although structurally belonging to the AT-type, 3-OST5 can thus functionally produce both AT- and gD- type modifications [194]. X-ray crystal structures for three 3-OSTs (3-OST-1, -3 and -5) have provided interesting insight into the substrate specificity of the different isoforms. They revealed, in particular, how gD-type enzymes select substrates containing IdoA2S through a lysine residue, whose absence in 3-OST-1 explains why this isoform does not act on IdoA2S containing substrates, but can indistinctly accommodate non-sulfated GlcA or IdoA residues [200,201,202]. The catalytic mechanism of 3-OST3 has also been studied using computational methods, which suggest how the protonation/deprotonation states of the catalytic residues (His186, Glu184 and Asp189) catalyse the transfer of sulfo groups from PAPS co-substrate to HS glucosamine units [203]. The expression of 3-OST genes appears to be tightly controlled in a spatiotemporal manner [204]. However, with very few exceptions, every cell line expresses at least one and generally more than one 3-OST. Therefore, every tissue can potentially display 3-*O-*sulfated HS, the regulation and function of which remain poorly understood [194].

In view of the large range of biological functions heparan sulfate controls, drugs inhibiting biosynthetic enzymes would be of great interest to target various pathologies such as inflammation, cancer and infection. The observation that O-sulfotransferases and kinases share related enzymatic mechanisms, feature similar structural elements in their active site and use very similar nucleotide donors of either activated sulfate or phosphate (PAPS and ATP, respectively) have motivated the screening of a library of kinase inhibitors against 2-OST [205]. Interestingly, known kinase inhibitors, including rottlerin and oxindole-based RAF kinase inhibitors were found to inhibit 2-OST in the middle µM range [206]. To be used as drugs targeting the HS biosynthetic pathways, such kind of molecules would have to be engineered to be more specific and to cross the plasma and the Golgi membranes.

## 6. Post Synthetic Mechanism Regulating HS Structure

Following biosynthesis, further modification of HS structure and function can take place at the post-synthetic level, through the action of enzymes targeting either HSPG protein core or HS chains. Major actors of such processes include heparanases, sheddases, and extracellular 6-*O-*endosulfatases of the Sulfs family.

### 6.1. Heparanase

Heparanase is an endo-β-D-glucuronidase that cleaves HS chains at the level of GlcA residues flanked by GlcNS. Consequently, cleavage of HS chains by heparanase results in the release of shorter, 10–20 sugar unit fragments exhibiting a NS-domain-like structure. A crystal structure of heparanase in complex with HS was recently solved and provided further insights into the substrate specificity of the enzyme [207]. This study showed that the heparanase catalytic cleft could accommodate a trisaccharide motif featuring *N-*S at position -2 and a 6-*O-*sulfate at position +1. Heparanase displays complex multi-faceted functions that take place in both intracellular and extracellular compartments. Initially secreted as an inactive pro-heparanase form, it binds to cell surface HSPGs to form a complex that will be endocytosed. Traffic from early endosomes to lysosomes leads to an activation of heparanase that will participate in the catabolism of HSPGs. Cleavage of HS chains by heparanase generates additional non-reducing saccharide extremities and facilitates the degradation process by exoglycosidases [208]. Lysosomal heparanase also participates in the formation of autophagosomes and can enter the nucleus where it can interact with the chromatin complex, regulating some histone methylation and gene transcription [209]. Furthermore, it can be secreted in the extracellular medium as an activated form that will degrade HS and induce a remodeling of the ECM. Extracellular activity of heparanase leads to the loosening of ECM architecture and the release of biologically active HS fragments as well as ECM stored HS ligands such as growth factors, chemokines and morphogens. Based on its multiple activities, heparanase has been associated with a wide range of physiological and pathological processes, including tumor growth, angiogenesis, invasion and chemoresistance, inflammation, fibrosis, coagulation and thrombosis or viral infection [208,210,211,212]. Noteworthy, heparanase II (Hpa2), a homolog of heparanase, has been identified [213]. HPa2 does not display heparanase enzyme activity, but is able to bind to HS, without inducing HSPG internalization. Hpa2 thus competes with heparanase and has been shown to act as a tumor suppressor [208].

### 6.2. Sheddase

Sheddase is the generic name given to enzymes that target the core protein of HSPGs, thereby leading to the release of their HS-bearing extracellular domains into solution. These include proteases, such as matrix metalloproteinases (MMPs), elastase, thrombin, granzyme B, ADAM and ADAMTS [214]. Cell-surface HSPGs and their shed ectodomains exhibit distinct biological activities. Shedding of HSPGs does notably take place during inflammation [215]. There, soluble ectodomains play major regulatory functions, by binding to inflammatory chemokines and thus affecting their bioavailability and organization of chemotactic gradients. On the one hand, they exhibit pro-inflammatory activities, as they have been shown to induce a toll-like receptor-4 dependent innate immune response [216], and to promote neutrophil migration [216,217,218]. On the other, they are also involved in the attenuation of the inflammatory response by down-modulating production of pro-inflammatory cytokines [219], and migration of immune cells [220,221,222]. Shedding of syndecan-1 has also been particularly studied in the context of cancer and has been shown to promote tumor cell proliferation and migration in vitro and tumor growth in vivo [223,224,225,226]. Surprisingly, shedding in myeloma tumors is enhanced by heparanase [227], thus illustrating the complex interplay between HS modifying post-synthetic processes. Finally, roles of HSPG shedding have been reported during bacterial pathogenesis [228]. Shedding of syndecan-1 is induced during *Staphylococcus aureus* infection [129], and has also been shown to enhance *Pseudomonas aeruginosa* virulence, possibly by inhibiting host defense mechanisms [229]. Similarly, it has been shown to promote *Listeria monocytogenes* infection by hindering host immune response [230].

### 6.3. The Sulfs

Along with the HS biosynthesis process, extracellular enzymes of the Sulf family can further regulate HS 6-*O*-sulfation by a unique post-synthetic mechanism. Sulfs are endosulfatases that were first identified in quails in 2001 [231]. Since 2001, orthologs have been identified in a mouse, rat, zebrafish and in humans, along with a second related enzyme: Sulf-2 [232].

Sulf-1 and Sulf-2 share common structural and functional features, which are largely dictated by a number of post-translational modifications. They are initially synthetized as pre-proteins that undergo a series of maturation processes, including the removal of the signal peptide and the cleavage of the polypeptide sequence by furin-type proteases, to generate a mature protein composed of two disulfide-bridged sub-units [233,234]. The 75 kDa N-terminal region is highly homologous between all Sulfs and exhibits the so-called catalytic domain (CAT), which comprises the enzyme’s active site, including the sulfatase strictly-conserved, post translationally modified N-formylglycine residue that is essential for the enzyme activity. The C-terminal region is essentially composed of a highly basic, hydrophilic domain (HD), which is a unique feature of the Sulfs. As for CAT, HD is a major functional domain of the Sulfs, as it binds with high affinity to HS substrates and it is required for the HS 6-*O*-endosulfatase activity of the enzyme [233,235]. Finally, the end of the Sulf C-terminal sub-unit is homologous to glucosamine-6-sulfatase (G6S), suggesting a role of this region in the recognition of glucosamine motifs. Additional post-translational modifications of the Sulfs include N-glycosylations, which account for ~20% of the protein’s molecular weight. Although the functional relevance of Sulf N-glycans is not fully understood yet, a study on QSulf-1 suggested that these were necessary for cell-surface localization and enzymatic activity [236].

Sulfs are unique amongst all other eukaryotic sulfatases, as they are the only members of this family to exhibit endo-enzyme activity. It has been recently proposed that the 6-*O*-desulfation of HS by the Sulfs occurs in a processive and orientated manner, starting from the non-reducing end of HS S-domains [237]. In this proposed model, CAT and HD play complementary roles to elicit the enzyme activity. The HD domain directs the recognition, binding selectivity and docking of the enzyme on HS substrates. In line with this, HD was shown to bind to heparin with a Kd in the nM range, leading to the formation of stable enzyme-substrate complexes [233,238]. This interaction was found to be more complex than a simple 1:1 binding model [238], as it most likely involves highly dynamic binding events involving multiple HS binding sites within HD [237], and exhibits atypical catch-bond type behavior, with increased lifetime when subjected to external forces [239]. Additional low affinity interactions involving HS binding epitopes within the CAT domain then contribute to the proper presentation of the polysaccharide to the enzyme active that catalyzes the 6-*O-*desulfation of glucosamine residues [238]. Loss of 6-*O-*sulfates finally reduces binding affinity, allowing processivity to take place.

Sulf substrate specificities are still poorly determined. They show a strong preference for [UA2S-GlcNS6S] trisulfated disaccharides units (UA stands for uronic acid), although studies also reported activity on [UA-GlcNS6S] disulfated disaccharides for quail and human Sulfs [237,240,241,242]. Noteworthy, such highly sulfated disaccharides units are mostly present within HS functional S-domains. Consequently, Sulfs constitute a unique regulatory mechanism of HS binding properties and functions. As such, Sulfs were initially discovered thanks to their ability to regulate Wnt signaling [231,241]. Wnt binds with high affinity to 6-*O-*sulfated HS, which acts as a negative regulator of the morphogen by preventing access to its cell-surface receptor, Frizzled. Remodeling of HS 6-*O-*sulfation pattern by the Sulfs results in a reduction in the binding affinity, enabling formation of a HS/Wnt/Fz functional complex [241]. Since, similar activation mechanisms have been reported for other HS-binding proteins, including GDNF [243] and BMP [244]. In contrast, another study also suggested that Sulf-catalyzed 6-*O-*desulfation of HS could have an inhibitory effect on Wnt, by facilitating its release and degradation [245]. These contradictory activities clearly exemplify the intricacy of HS regulation by the Sulfs. Sulfs also modulate the activity of a number of growth factors using HS as a cell-surface receptor/co-receptor. These include FGF1 [237,246], FGF2 [237,247,248,249], HGF [249,250], HB-EGF [248,251], amphiregulin [252] or TGFβ [253]. Amongst these, a noteworthy example is that of FGF-2. For FGF-2, the 6-*O-*sulfation of HS is not required for high affinity binding, but is necessary for the formation of a FGF/FGFR/HS ternary complex capable of triggering cell signaling [254,255]. Sulfs could thus finely tune FGF-2 activity by catalyzing the rapid conversion of 6-*O-*sulfated FGF-2, promoting HS into inhibitory motifs that fail to induce a cell response, but are still able to bind and sequester the growth factor. Finally, Sulfs have been reported to decrease the ability of heparin to bind to chemokine CXCL12 in vitro [246]. As many chemokines require 6-*O-*sulfates to efficiently bind to HS, it could be anticipated that Sulfs may modulate other chemokine/HS interactions, with important functional consequences for chemokine storage, protection against proteolytic degradation migration or oligomerization [256].

Through their ability to alter HS/protein interactions, Sulfs have been linked to many physiopathological processes, including development, tissue homeostasis/regeneration and cancer. The importance of Sulfs during development has been highlighted in many studies using Sulf-knockout animals, with reported roles during neuronal development [257,258,259,260], skeletal and cartilage development [261,262], formation of the inner ear [263] and dentinogenesis [264]. Mice deficient in both Sulf-1 and Sulf-2 displayed high neonatal mortality and severe morphological abnormalities, including renal, lung, skeletal, and neuronal defects [257,262,265,266]. However, single knockout of either Sulf-1 or Sulf-2 resulted in relatively mild phenotypes, suggesting overlapping/redundant functions of the two Sulf forms during development. In contrast, major differences between Sulf-1 and Sulf-2 have been reported in the context of cancer. Sulf-1 has been mostly associated with anti-oncogenic effects, while Sulf-2 exhibits pro-oncogenic activities [16,267]. It is still unclear how two closely related enzymes with nearly identical activities in vitro can exhibit such antagonistic properties during tumor development. However, differences in Sulf-1/Sulf-2 substrate specificities, levels of expression and/or cell-surface/ECM distribution could be hypothesized.

Despite increasing interest as regulators of HS activities, Sulfs remain poorly characterized enzymes. In this context, the major challenges ahead will be to get further insights into Sulf complex structural organization, to clarify the structural and functional features discriminating Sulf-1 and Sulf-2 forms and to investigate the role of the Sulfs in new fields in which HS are actively involved, such as inflammation or neurodegenerative diseases.

## 7. Conclusion and Future Directions

The biosynthesis of HS and the assembly of defined and functional protein binding sites are the results of a complex pathway involving backbone elongation, followed by several modification steps. Many of the enzymes involved in this process have been studied, both in vitro and in vivo. Whereas HS biosynthesis has been traditionally described as a sequential process, wherein the EXT, NDST, C5-epi, 2-, 6- and 3-OST come into action one after another, the analysis of these enzymes’ substrate specificities gave rise to a more intricate figure.

Considerable efforts have been made to produce chemically defined oligosaccharides [268], enabling the solving of the structure of the enzymes, in an apo- and a glycan bound- form. Such data permitted the identification of the molecular determinants that the enzymes used to distinguish the epimerization/sulfation patterns of their substrates. For example, the C5-epi requires a GlcNS residue upstream of its targeted GlcA/IdoA and thus acts after the NDST. The 2-OST can sulfate both GlcA and IdoA, and can thus intervene before or after the C5-epi, the latter being strongly favored, presumably as these two enzymes can form a complex [123,177]. The targeted residue, however, should be flanked both at the reducing and non-reducing sides by GlcNS, indicating that NDST activity necessarily precedes that of 2-OST [187]. In addition, 6-OSTs have a relatively broad substrate specificity, sulfating both GlcNAc and GlcNS [269]. NDSTs, which catalyze the GlcNAc conversion into GlcNS, intervene shortly after the polymerization and the resulting S-domains are the seat for most of the above-mentioned modifications.

The preponderance of each of these enzymes or enzyme isoforms, within a given tissue or cell, will affect the structure of the final product but also the order in which the modifications occur, as observed in knockdown or overexpression experiments. Overexpression of NDST1 or NDST2 leads to an increase in *N-*sulfation, as expected, without modifying epimerization and *O-*sulfation levels. Intriguingly, however, this correlated with an increase in the average chain length, suggesting a coupling between the EXT-mediated polymerization and the *N-*sulfation processes. This observation can be explained by a decrease in K_m_ for GlcA-transfer activity of the polymerase, which recognizes better *N-*sulfated substrates at the penultimate GlcN residue, towards the non-reducing end [147]. C5-epi overexpression also results in increased chain length through an unknown mechanism [270]. Furthermore, 2-OST overexpression was shown to diminish the IdoA content of HS. At high concentration, this enzyme, which normally preferentially targets IdoA, will start to increase the 2-*O-*sulfation of GlcA residues, which are then no longer substrate for the C5-epi and thus cannot be converted back to IdoA [271]. Overexpression of 6-OSTs increased both GlcNS and GlcNAc 6-*O-*sulfation, but also reduced the total IdoA content, 2-*O-*sulfation and modified the distribution of the *N-*sulfated domains [272]. HS produced by C5-epi and 2-OST knockout animals are enriched in *N-* and 6-*O-*sulfation, which could suggest a compensation mechanism to overcome the loss of IdoA and 2-*O-*sulfate, but the underlying mechanism remains unknown [127,179].

All together, these data indicate that HS chain structure results from multiple combinatorial possibilities generated by a variety of either sequential or competing acting enzymes, the balance of which depending in part on their expression level. The availability of the biosynthesis precursors, such as UDP-sugars and the sulfate donor PAPS, as well as their transporters [146,273], needs also to be taken into account in order to have an integrated view of the overall mechanism.

**Figure 3:**
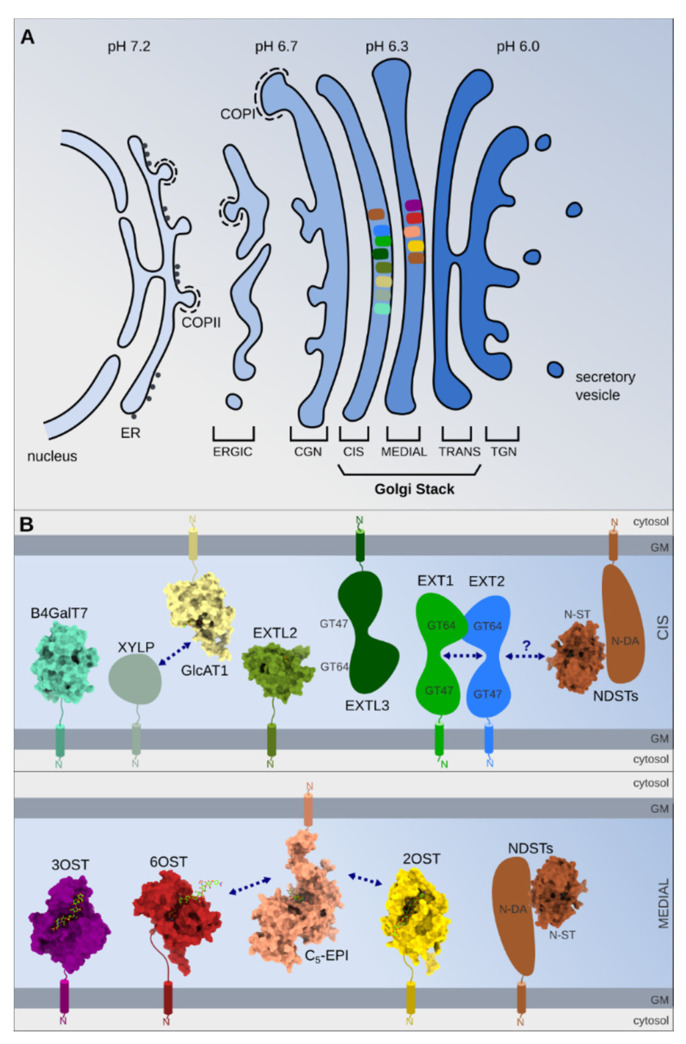
(**A**) Schematic illustrating the Golgi complex. COPII-coated vesicles ensure the protein transport from the endoplasmic reticulum (ER) to the vesicular clusters (ERGIC), which fuse to form the cis-Golgi network (CGN). The Golgi stack is constituted by three separate compartments: the cis-, medial- and trans-cisternae. The last Golgi element, facing the plasma membrane is the trans-Golgi network (TGN). The color change of the ER, ERGIC and Golgi stack reflect the pH gradient. Some enzyme localizations are schematically depicted by different colors corresponding to enzymes represented in part B. Golgi compartmentalization of these enzymes was suggested by using confocal immunocytochemistry and different Golgi markers or Golgi drug treatments (nocodazole, brefeldin A). (**B**) Schematic domain composition of the enzymes involved in HS biosynthesis. Proteins, for which an X-ray crystallography structure has been reported, are shown in surface representation and bound ligands in stick representation. PDB-IDs (B4GalT7: 4IRQ, GlcAT1: 1V84, EXTL2: 10N8, NST: 1NST, 3OST: 3UAN, 6OST: 5T0A, 2OST: 4NDZ, C5-EPI: 6I01). Arrows indicate reported interactions between proteins.

Finally, the enzyme subcompartmentalization, their dynamic and possible interactions within the Golgi apparatus, also represents key aspects of HS structure determination and regulation (Figure 3).

As shown using GFP-fused enzymes combined with fluorescence microscopy, NDST1, C5-epimerase and 2-OST colocalize with a medial-Golgi marker [274], whereas the polymerases EXT1, EXT2, and the sulfotransferases HS6STs are located in the cis-Golgi compartment [275,276]. Pharmacologic treatments, such as nocodazole, that break up the Golgi apparatus into functional ministacks or brefeldin A, which causes the fusion of Golgi cisternae with the ER compartment, induce differential enzyme relocalisation [276]. Using xyloside analogs, which are readily recognized as primers by the GAG biosynthesis machinery, resulted in the generation of distinct population of GAG chains, suggesting that the specific aglycone moieties of these analogs could dictate their localization in different Golgi sub-compartments where they would encounter different HS biosynthesis enzymes [277]. Further studies, preferentially using endogenous and native rather than over-expressed and fused enzymes, will be needed to understand and determine their accurate Golgi distribution. Surprisingly, a recent study reported that the HS modifying enzyme 3-OST-2 can be distributed to the cell surface, possibly associated with syndecan-2, where its role is unknown [128]. Similarly, 6-OST was shown to be secreted, in an active form, in the medium of cultured cells [278].

Some of the enzymes appear to form complexes, the assembly of which has been proposed to also impact the final HS structure [279].

The 2-phosphoxylose phosphatase, which dephosphorylates the Xyl residue and regulates the formation of the linkage region, interacts with the glucuronyltransferase-I, the enzyme catalyzing the addition of the fourth sugar unit (GlcA) of the linker [136]. The polymerases EXT1 and EXT2 form a complex in the ER, which appears to be necessary for Golgi translocation where it is functionally active [140].

Based on the observation that over-expression of EXT2 increases both NDST activity and protein content in cells, while over-expression of EXT1 has the opposite effect, it was speculated that NDST1 could depend on EXT2 for its transport to the proper Golgi compartment. In such model, EXT1, in binding to EXT2, would compete out NDST1 from the complex. Supporting this view, co-immunoprecipitation experiments showed association between EXT2 and NDST1 [169]. EXTL3 also controls the sulfotransferase activity of NDST1 by interacting with this enzyme, which contributes to the generation of unsubstituted GlcNH3+ residue [167]. The physical interaction between C5-epimerase and 2-OST has also been described as necessary for their translocation and proper localization in the Golgi [123]. Direct binding has been demonstrated at the biochemical level and it was observed, in vitro, that the complex generates extended sequences of contiguous IdoA2S-GlcNS disaccharides, adopting a processive and concerted catalytic activity. In contrast, when the two enzymes work separately, epimerization and sulfation are randomly introduced along the HS chain [177]. C5-epi has also been shown to interact with 6-OST, although to a lesser extent than with 2-OST, but the functional consequences of such binding have not been determined [175].

Given the complex interplay of enzymes involved in HS biosynthesis, many open questions remain. It has been suggested that, as described above, enzymes associate with each other to form specific “nanomachines” committed to HS motif assembly, the catalytic output of which depends on their stoichiometry and organization mode. Physical association of these enzymes would provide a mechanism for channeling substrates between enzymes and explain the clustered modifications along the chain (S domains). The assembly of enzymes into a so-called GAGosome complex has thus been a popular model to explain findings, but its existence is still under debate. In most cases, complexes were identified following co-immunolocalization or co-immunoprecipitation of proteins from cell extracts, which could be mediated by substrates, a still unknown scaffold protein or other cellular components. To resolve the existence, the composition and the function of such supramolecular complexes, further in depth biochemical characterization of the GAG biosynthesis enzymes would be needed. Tissue-specific expression of the various enzyme isoforms may account for the structural variability of HS; however, in many cases no obvious correlations have been found between the level of expression of the individual enzymes and the structure of the resulting HS chains, and how this differential expression would affect the organization of the complex is also unknown [123,140,169,275,280]. Interestingly, it has been shown that various Golgi-localized glycosidases and glycosyltransferases form heteromeric complexes, depending on Golgi acidity [281]. A pH gradient characterizing the ERGIC to TGN [282] could therefore trigger the formation of different complexes of HS biosynthesis enzymes as they traffic through the different Golgi sub-compartments.

It becomes clear that, even having a complete knowledge of the expression levels of all the relevant gene products, we do not still understand enough about the structure and pathways, the relationship and the dynamic of the involved molecules, to predict the precise HS structures elaborated by a given cell type, under a given condition. Exciting developments at the interface between structural biochemistry and integrated cellular biology will be key to reveal how the variable and dynamic nature of the gagosylation machinery can generate such a large biological diversity and complexity.

## Figures and Tables

**Table 1:** Functions and localizations of HS biosynthetic and extracellular modifying enzymes.

Enzymes	Function	Golgi/Cell Localization [Reference]
XylT-1/XylT-2 GalT-1/GalT-2 GlcAT-1	Formation of linkage region	Cis/Medial [124] Cis/Medial [123] Cis/Medial [123]
EXTL EXT1/EXT2	GlcNAc addition HS Elongation	Not yet specified Cis [125,126]
NDSTs	GlcNAc *N*-deacetylation/N-sulfation	Cis/Medial [123]
C_5_-Epi 2OST	Glc C5 Epimerization GlcA/IdoA 2-*O*-sulfation	Medial [123] Medial [123]
6OSTs	GlcN 6-*O*-sulfation	Cis/Medial [127]
3OSTs	GlcN 3-*O*-sulfation	Cis/Cell surface * [128]
Sulf-1/Sulf-2	GlcNS6S 6-*O*-desulfation	Cell surface [129]

Enzymes localizations have been determined using confocal immunocytochemistry, over-expressed fusion proteins, and different well-known Golgi markers or Golgi drug treatments. These localizations might thus not exactly reflect the localization of the endogenous proteins. * The HS modifying enzyme 3-OST-2 has been detected outside of the Golgi apparatus, at the plasma membrane, where it co-localizes with syndecan-2.
